# Low-Grade Inflammation Associated with Major Depression Subtypes: A Cross-Sectional Study

**DOI:** 10.3390/brainsci14090850

**Published:** 2024-08-23

**Authors:** Veronique Bernier, Ghada Alsaleh, Camille Point, Benjamin Wacquier, Jean-Pol Lanquart, Gwenolé Loas, Matthieu Hein

**Affiliations:** 1 Department of Psychiatry and Sleep Laboratory, Erasme Hospital, Université Libre de Bruxelles—ULB, 1070 Brussels, Belgium; 2 Botnar Research Centre, Nuffield Department of Orthopaedics, Rheumatology and Musculoskeletal Sciences (NDORMS), University of Oxford, Oxford OX3 7LD, UK; 3 Laboratoire de Psychologie Médicale et Addictologie (ULB312), Université Libre de Bruxelles—ULB, 1020 Brussels, Belgium

**Keywords:** low-grade inflammation, C-reactive protein, major depression, atypical depression subtype, polysomnography

## Abstract

Major depressive disorder (MDD) is associated with inflammation and a high level of comorbidities. Atypical depression (AD) is a MDD subtype based on DSM criteria, that could have specific underlying biological mechanisms. AD is associated with elevated cardiovascular (CVD) comorbidities, higher risk of suicide attempts, hypersomnia, and anxiety disorder. In this study, we aim to investigate if AD and polysomnographic parameters could be associated with low-grade inflammation (LGI). LGI is defined by a range from 3 to 10 mg/L of C-reactive protein levels. We carried out a retrospective cohort study in which 765 individuals with MDD were split into two groups: with and without LGI. Our results exhibit differences between the groups for the polysomnographic parameters, with the LGI group showing parameters already associated with inflammation such as reduced rapid eye movement sleep and elevated hypoxemia markers (identified as CVD risk factor). We found that AD is associated with LGI (OR 1.48; *p* = 0.047) after adjustment. Likewise, we found an LGI prevalence in AD higher (34.8%) than in MDD without atypical features (26.8%). Overall, these results confirm the low-grade inflammation feature of AD and highlight polysomnographic parameters associated with LGI that could also act as risk factors in this context.

## 1. Introduction

Depression is a psychiatric disorder that includes several subtypes differing in diagnosis criteria, severity, and treatment. Depression is affecting an estimated 280 million people in the world (5%) and mental disorders are now the leading cause of disability worldwide [1,2]. The diagnosis of major depressive disorder (MDD) is based on the Diagnostic and Statistical Manual of Mental Disorders (DSM-5) [3]. MDD is defined by a minimum of five out of nine of the following criteria: anhedonia, depressive mood, weight variation, sleep disturbance, alteration of psychomotor activity, tiredness, feelings of guilt or worthlessness, suicide attempts, and cognitive disturbance.

MDD is associated with inflammation: about 27% of individuals suffering from MDD exhibit low-grade inflammation (LGI) [4]. LGI is a chronic inflammation defined by a C-reactive protein (CRP) level that ranges from 3 to 10 mg/L [5]. Inflammation could increase the risk of developing depressive episodes whereas conventional antidepressants seem to be ineffective against inflammation [6,7,8]. For depressive patients, inflammation is also associated with a worse prognosis including treatment resistance, increased suicide attempts, symptom enhancement, and relapse [9,10,11,12]. Inflammation could also increase the risk of developing cardiometabolic comorbidities that are frequent in this pathology such as type 2 diabetes or cardiovascular diseases (CVD) [13]. LGI is already identified as a CVD risk factor by the American Cardiology Association. 

The biological mechanisms that could explain the deleterious effect of inflammation in depression are numerous and not completely defined. One of the hypotheses is the sickness behavior syndrome described first in the works of Dantzer [14]. During an infectious episode, peripheral inflammation could stimulate the central nervous system (CNS) via the nervous (vagus nerve) and humoral (pro-inflammatory cytokines) pathways through more permissive regions of the blood–brain barrier. This could in turn lead to the production of pro-inflammatory cytokines at the central level and trigger sickness behavior (SB) syndrome [15,16]. SB exhibits somatic/vegetative symptoms such as fatigue, sleep impairment, abnormal appetite, and psychomotor retardation. It also induces behavioral changes like depressed mood, anhedonia, and cognitive dysfunction. The clinical evidence of SB is shown with the interferon alpha treatment used in cancer or hepatitis C [17]. Most treated patients experience somatic/vegetative symptoms and among them 30% to 45% exhibit psychological symptoms [17]. Interestingly, psychiatric assessments performed before the treatment show that psychological symptoms occur preferentially in more fragile individuals due to environmental reasons or genetic inheritance [17,18]. The overlap between the neurovegetative and psychological symptoms of SB with those of depression as described in DSM criteria, could indicate common biological pathways. But in case of an infectious or inflammatory episode when the inflammation ends, so do the symptoms. On the contrary, for depression, a chronic state of inflammation could lead to supporting psychological and neurovegetative symptoms and therefore, act as a risk factor in this context. Moreover, low-grade inflammation could be sufficient to act in vulnerable individuals [18]. The criterion of vulnerability is essential in this context; depression is first and foremost a multifactorial disease where environmental factors and/or genetic determinants are likely presumed to act. Another explanation could be linked to treatment. Studies show that the level of CRP could modulate the efficiency of antidepressant types. Selective serotonin reuptake inhibitors (SSRI) could act more efficiently for a CRP level under 1 mg/L while tricyclics or SSRI–bupropion would be more efficient for a CRP level over 1 mg/L [19,20]. More studies are needed to confirm this, but cytokines could therefore sabotage the effect of front-line treatments such as SSRI. Finally, it has been shown more recently that peripheral inflammation could be associated with the alteration of brain structure involved in emotional regulation, award processing, and cognitive control in depression [21].

In this study, we focus on MDD with atypical features (AD), which is a subtype of major depressive disorder, with an estimated prevalence of 15–29% [22]. The diagnosis of AD is based on DSM-5/IV-TR criteria that are mood reactivity and two others: hyperphagia, leaden paralysis, hypersomnia, and interpersonal rejection sensitivity. AD could have different underlying biological mechanisms than MDD without atypical features (OD) [23,24]. Thus, melancholic depression type is associated with hyperactivation of the hypothalamic–pituitary adrenal axis (HPA) and an elevated cortisol level [24]. In contrast, AD has been shown to be associated with a hypoactivation of the HPA axis and elevated inflammatory markers [24,25,26]. AD is associated, independently of inflammation, with a higher incidence of metabolic syndrome, elevated waist circumference, and fasting glucose level [27,28]. There is also a robust consensus that AD is associated with a higher cardiovascular risk than OD [29]. However, LGI which is already identified as a CVD risk factor as aforementioned, has either not been investigated or investigated with a limited population in AD [30,31,32]. 

Another distinctive criterion of AD is hypersomnia. Impaired sleep is frequent in depression and could be at the same time a diagnosed criterion and a risk factor [33]. Moreover, sleep disturbance is associated with elevated inflammatory markers and could act in this context [34]. 

In this study, we aim to investigate the impact of MDD subtypes (AD/OD) on the occurrence of LGI in a large cohort of patients suffering from major depression. We also aim to detect any difference in the polysomnography between both groups: with and without LGI. Our first research hypothesis is that the prevalence of AD is higher than MDD without atypical features in the LGI group. Our second research hypothesis is that differences in polysomnographic parameters could occur between both groups as well. Finally, we aim to propose a model of the AD pathophysiological environment to improve our understanding of this depression subtype and to identify future research.

## 2. Materials and Methods

### 2.1. Population

This study was approved by the Hospital and Medical School Ethics Committee of the Erasme Hospital (Brussels University Hospital) (reference P2023/573—approval date: 16 January 2024) in compliance with the recommendations of the Declaration of Helsinki.

A total of 765 individuals diagnosed with major depression having performed a polysomnographic recording between 1 January 2002 and 31 December 2020 were recruited from the clinical database of the Erasme Hospital Sleep Laboratory. These individuals were referred to the sleep laboratory by physicians specialized in sleep medicine after an outpatient consultation. This first consultation aimed to determine an initial diagnostic hypothesis and to research the presence of comorbid sleep disorders.

Inclusion criteria: age ≥ 18 years and the presence of a major depressive episode meeting DSM criteria (DSM IV-TR before 2013 and DSM-5 after 2013) [3,35]. According to the DSM, major depressive disorder is defined by a minimum of five out of nine of the following criteria: anhedonia, depressive mood, weight variation, sleep disturbance, alteration of psychomotor activity, tiredness, feelings of guilt or worthlessness, suicide attempt, and cognitive disturbance. One of the first two at minimum is required. These criteria are qualitative but also quantitative, they must possess characteristics of frequency and duration. We included individuals with or without treatment. The treatment could be either benzodiazepines or antidepressants or a combination of both.

Exclusion criteria: The presence of psychiatric disorders other than major depression. In order to avoid physiopathology or inflammatory biases, we exclude severe somatic pathology, infectious or inflammatory disease, a CRP level > 10 mg/L, pregnancy, central hypersomnia, sleep apnea syndrome with central predominance, obstructive sleep apnea syndrome already known or being treated before entering the sleep laboratory, current or past head trauma, current or past injury to the central nervous system affecting the respiratory centers, craniofacial or rib cage malformations and substance abuse.

### 2.2. Medical and Psychiatric Assessment of Participant

A medical interview and somatic assessment (including blood test, electrocardiogram, daytime electro-encephalogram, and urinalysis) were systematically carried out in all these patients during their admission to the hospital to diagnose their potential somatic comorbidities.

The CRP level is measured by immuno-turbidimetry on plasma. The material is a Roche CRP4-Cobas with a minimum detection level of 0.3 mg/L. We excluded individuals with CRP levels ≥ 10 mg/L to avoid biases that may be induced by infections or inflammatory diseases. Subsequently, based on these CRP levels, low-grade inflammation is defined as absent when CRP levels are <3 mg/L and as present when CRP levels were ≥3 mg/L [36]. 

A systematic psychiatric evaluation was performed by a psychiatrist from the unit on all these patients to diagnose their potential psychiatric comorbidities (including subtypes of major depression) according to the above-mentioned DSM diagnostic criteria. Finally, all these patients completed a series of 3 self-questionnaires to enable an initial assessment of their subjective complaints of depression, daytime sleepiness and insomnia: the Beck Depression Inventory (13_itemBDI), the Epworth Sleepiness Scale (ESS) and the Insomnia Severity Index (ISI) [see Supplementary Data Section S1.1 for more details].

### 2.3. Sleep Assessment and Examination 

Sleep history. This interview was conducted by a sleep laboratory psychiatrist to assess the complete inventory of the individual self-reported sleep complaints including sleep habits, insomnia, sleep apneas, abnormal nocturnal movement, and restless syndrome [see Supplementary Data Section S1.2 for more details].

Polysomnography. Participants have benefited from a polysomnographic recording from which data are collected for analysis. The polysomnography is compliant with the recommendations of the American Academy of Sleep Medicine [37] [see Supplementary Data Section S1.3 for more details].

### 2.4. Statistical Analyses

Statistical analyses were carried out using Stata and SPSS version 29.0.2.0 [38,39]. To conduct our analyses, we divided our sample of MDD into a control group without LGI (CRP level < 3 mg/L) and a patient group with LGI (CRP level ≥ 3 mg/L). Categorical data are described by percentages and numbers, while continuous variables are described according to their asymmetrical distribution by their median and their percentile 25–75. According to the data distribution we performed Wilcoxon non-parametric test and for the dichotomously distributed data we used the Chi-square test.

Univariate logistic regression models were used to identify the correlates of depression subtypes (categorized: MDD with and without atypical features) including LGI and potential confounding factors. Following a review of the literature on factors associated with LGI, potential confounders included in this study were body mass index (BMI) (categorized: <25 kg/m^2^, ≥25 kg/m^2^), age (categorized: <40 years, ≥40 years), sleep movement disorders (categorized: absent, moderate to severe periodic limb movement syndrome alone, restless legs syndrome alone or combined with periodic limb movement syndrome), insomnia disorders (categorized: absent, insomnia without short sleep duration, insomnia with short sleep duration), severity of obstructive sleep apnea syndrome (OSAS) (categorized: absent, with TO_2_ 90% < 10 min, with TO_2_ 90% ≥ 10 min), depression severity (categorized: mild to moderate, severe) and as binary variables: gender, antidepressant therapy, benzodiazepine receptor agonists, smoking, alcohol consumption, caffeine consumption, excessive daytime sleepiness, hypertension, dyslipidemia, type 2 diabetes, cardiovascular comorbidities and aspirin therapy [5,40,41,42,43,44,45,46,47,48,49].

In order to adjust our results concerning the polysomnographic variables for antidepressant therapy and benzodiazepine receptor agonists, we used multivariate quantile regression models (based on the median). In these models, polysomnographic data were considered as the dependent variables whereas antidepressant therapy and benzodiazepine receptor agonists were used as predictors to adjust the regression coefficient between the two groups of individuals with major depressive disorder. We decided to adjust our results concerning the polysomnographic parameters by antidepressant therapy and benzodiazepine receptor agonists as psychotropic drugs can have a significant impact on sleep architecture [50,51]. 

In multivariate logistic regression models, the risk of LGI associated with depression subtypes was adjusted for significant confounding factors identified during the univariate analyses. These selected factors were any that showed a significant difference between both groups (i.e., *p* < 0.05). In the multivariable analysis, the variables were introduced hierarchically (models 1, 2, 3, and 4) to facilitate the clarity of the results and the clinical interpretation.

For calculation of LGI estimated prevalence in AD and OD, we corrected both groups (with and without LGI) by considering a range strictly above 3 mg/L of CRP level instead of ≥3 mg/L. We made this adjustment to be consistent with the LGI value range defined in the Cambridge meta-analysis [4].

The aim of the Principal Components Analysis (PCA) is to reveal systematic covariations among a group of variables. The PCA variance is a measure of how much of the total variance in the original dataset is explained by each principal component. PCA uniqueness represents the variance that is “unique” to the variable and not shared with other variables. The PCA Varmix analysis was performed with SPSS version 29.0.2.0, the data, and JASP software version 0.19.0 for the diagram [39]. 

Results were considered significant when the *p*-value was <0.05.

## 3. Results

### 3.1. Polysomnography 

After adjustment for the treatment, the polysomnographic analyses showed a significant difference between both groups with and without LGI in 25% of the criteria (i.e., 4 criteria out of 16) [Table 1]. The results show an increase for rapid eye movement sleep (REM) (*p* = 0.006) and on the contrary a decrease in REM latency (*p* = 0.024). An increase in the Oxygen Desaturation Index (ODI) (*p* < 0.001) and the total time under 90% of oxygen saturation (SaO_2_) (*p* = 0.001). ODI and SaO_2_ are hypoxemia markers, which means a potential deficiency of oxygen reaching the tissues. 

### 3.2. MDD Groups (LGI, No-LGI) 

#### 3.2.1. Univariate Analyses

Overall, the LGI group is mainly female, aged more than 40 years old, overweight (BMI 30.5 (25.6; 36.2)) have dyslipidemia (51.1%) and hypertension (46.3%). The LGI group also has more AD (27.7%), type 2 diabetes (17.6%), CVD (12.3%), and OSAS (22%) than the non-LGI population (Table 2). In our sample no individuals treated with the monoamine oxidase inhibitor (MAOI) treatment were present.

#### 3.2.2. Multivariate Analyses 

The multivariate analyses are carried out considering the significant parameters of the univariate analyses [Table 2]. These results show that AD is associated with a higher risk of LGI with an OR 1.48 [CI 95% 1.01; 2.18] after adjustment of the main confounding biases (gender, age, BMI, smoking status, dyslipidemia, hypertension, type 2 diabetes, CVD, and OSAS) [Table 3].

#### 3.2.3. The PCA Analysis 

**Figure 1:**
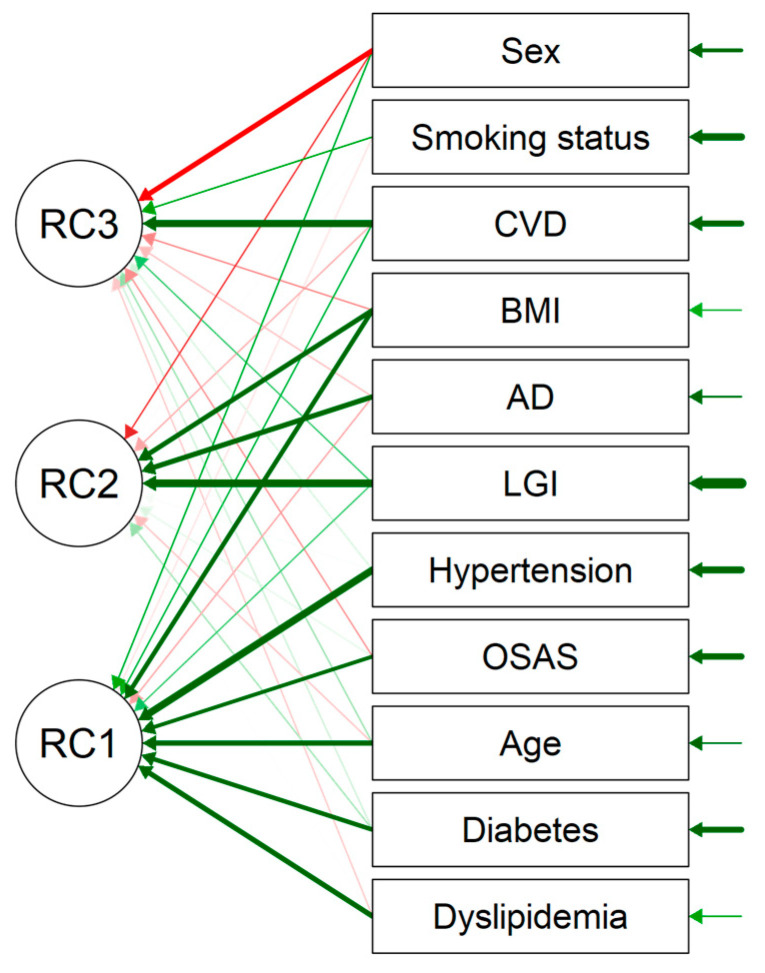
Principal component analysis (PCA). RC1, 2, and 3 are the axes that contain the correlated variables. Green arrows show positively correlation, and red arrows show a negatively correlation. The thickness of the arrows shows the strength of the correlation. Chi-squared test < 0.001.

The Principal Components Analysis (PCA) reveals systematic covariations among a group of variables. Figure 1 and Table 4 show that on the first axis (RC1) the main cardiometabolic risk factors: BMI, age, OSAS, diabetes, hypertension, and dyslipidemia are correlated [Figure 1, Table 4 and Table 5]. The LGI is also present on the RC1 axis but with a minimal loading of 0.235 [Figure 1, Table 5]. On the second axis (RC2) AD is positively correlated with LGI and BMI [Figure 1, Table 5]. On the third axis (RC3) the CVD is negatively correlated with sex (male) and positively with the smoking status as well as LGI but with a minimal loading of 0.246 [Figure 1, Table 5]. All the principal components retained in the analysis explain 43.9% of the total variance of the data set [Table 4].

#### 3.2.4. Estimated Prevalence Calculation 

The estimated prevalence of LGI in AD is 34.8% and is higher than in OD (26.8%) (*p* = 0.047) [Table 6]. The AD group shows a higher tendency (*p* = 0.064) to hypertension than the OD group [Table 7].

## 4. Discussion

The polysomnographic data analyses showed 4 out of 16 parameters different between LGI and non-LGI groups. The concerned criteria are % of rapid eye movement sleep (REM), REM latency, and 2 hypoxemia markers. REM is related to the sleep stage in which most dreams occur. The REM latency refers to the amount of time between the onset of sleep and the first REM stage. REM sleep is shorter and with a higher latency in LGI individuals. Few studies have investigated this criterion but in animal model patterns it has been shown that a reduced REM is associated with elevated inflammatory markers [52]. Nevertheless, antidepressants could impact the %REM/REM latency, and the LGI group shows a tendency of higher treated MDD patients (45.4%, *p* = 0.076) than non-LGI (38.5%) [Table 2] [50,53]. Therefore, treatment could also contribute to explaining this result.

The hypoxemia criteria (oxygen desaturation index and total time under 90% of SaO_2_) are more noticeable in the case of LGI individuals. Both markers are presumed to estimate the severity of obstructive sleep apnea syndrome (OSAS) [54,55]. OSAS is frequent with a prevalence of 1/7 of the adult population worldwide [56]. OSAS induces stress in the cardiovascular system and is associated with inflammation [55]. OSAS is also a risk factor for cardiometabolic disease [55,57,58,59]. Thus, the criteria that show a difference between both groups (with and without LGI) could be consistent with an inflammatory context and/or a higher cardiovascular risk. 

The results of the univariate analyses (Table 2) highlight the cardiometabolic features (dyslipidemia, hypertension, overweight) of the LGI group that are consistent with a higher rate of diabetes, CVD, and OSAS than the non-LGI group. The proportion of female, and middle-aged (and older) people is also higher in the LGI group. This result is in line with depression in general which first affects females, and then middle-aged and elderly people [60].

As we presumed, the odds ratio of AD in the LGI group is higher than in the non-LGI group (1.57 [95% CI: 1.09; 2.24]) and remains significantly more elevated after adjustment of all the confounding biases selected from the univariate analyses (OR 1.48 [95% CI: 1.01; 2.18]). The principal component analysis is consistent with this result and confirms that AD is correlated with LGI (RC2) [Figure 1, Table 5]. Interestingly, the AD uniqueness that represents the variance “unique” to the variable is high (0.624) and all the principal components explain 43.9% of the global variation. These results are consistent with a psychiatric context where psychological issue is often the main risk factor.

Likewise, to compare with the literature background [4], we precisely adjusted the calculation of AD LGI prevalence to a range above 3 to 10 mg/L of CRP level. As expected, we found a higher atypical depression LGI prevalence (34.8%) than non-atypical depression subtype LGI prevalence (26.8%, *p* = 0.047) (Table 6). Interestingly, the 26.8% prevalence of the non-atypical subtype depression LGI group is close to the LGI value of all MDD types (27%) from the Osimo meta-analysis, while the atypical depression LGI level is higher than both [4]. This result could perhaps contribute to explaining the severity of the AD subtype prognosis. Thus, these results confirm the association between AD and inflammation. They also specify both the range of inflammatory markers involved and the level of risk.

Another diagnosis criterion of AD, hyperphagia related to increased appetite, could also play a role. It has been shown that AD is associated, independently from inflammation, with increased waist circumference, elevated fasting glucose, and metabolic syndrome [27]. In a previous study, AD has been associated with a specific diet pattern the Western diet (WD) [61]. The WD is a high-fat, high-sugar, low protective nutrients diet [62,63]. The WD is rich in ultra-processed food, refined carbohydrates, and simple sugars like sucrose or glucose syrup that have the capability to dramatically increase glycaemia [63,64]. The hyperglycemic criterion of WD emphasizes its obesogenic feature [63]. Ultra-processed food also has high palatability and reduced satiety effect that led to increase the food intake [65]. Thus, an increased appetite (AD symptom) added to the WD pattern could contribute to increased waist circumference, overweight, and metabolic issues (dyslipidemia, metabolic syndrome), associated with AD. Moreover, in our results, hypertension tends to be higher in AD vs. OD (*p* = 0.065) and could consequently enhance the cardiometabolic feature of this subtype [Table 7]. Finally, the WD is a pro-inflammatory diet pattern associated with elevated CRP levels and could act as a risk factor in the AD inflammatory context as well [66,67,68,69].

From a clinical perspective, monoamine oxidase inhibitor (MAOI) drugs are presumed to be more effective in AD than conventional treatment. Interestingly, MAOI could have anti-inflammatory properties that could perhaps contribute to explaining this result [55]. However, MAOI has also severe side effects and strict diet restrictions [70]. Foods rich in tyramine-type amino acids (e.g., certain vegetables, cheeses, or meats) should be avoided to prevent a serious hypertensive crisis. Due to this concern and despite improving formula MAOI is still under-prescribed as first-line treatment and was not present in our sample [70,71].

**Figure 2:**
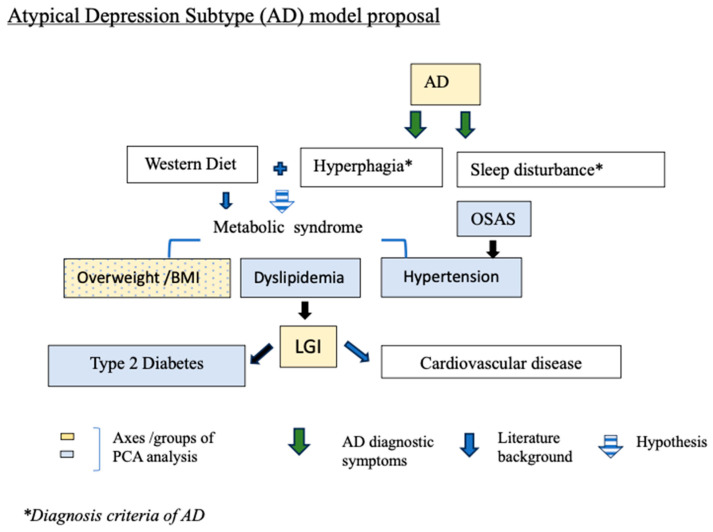
AD underlaying cardiometabolic risk factors. The colored square groups 1 and 2 (blue and yellow) correspond to the 2 axes of the PCA analysis (Figure 1: RC1 and RC2). Group 1/RC1 (square blue): OSAS, hypertension, dyslipidemia, type 2 diabetes, and BMI; Group 2/RD2 (square yellow): AD, BMI, LGI. The green arrows are AD diagnostic symptoms DSM criteria and the blue arrows are the literature background.

Overall, the results show that AD is associated with LGI. Furthermore, some of its diagnosis criteria such as sleep impairment but also nutrition could act as risk factors in this context (Figure 2). LGI, which is already a CVD risk factor could contribute to explaining the high level of cardiac comorbidities of the AD depression subtype.

In Figure 2, we propose a model of the AD inflammatory context. The symptoms of this subtype (hyperphagia and sleep impairment) associated with an unhealthy diet pattern (the Western diet) could contribute to explaining the LGI prevalence and ultimately its cardiometabolic context. This model is based on the DSM criteria of AD but also on the correlation revealed in our PCA analysis and, for the Western diet, the literature background [61].

Limits of the study. The first limit is reliant on the sourcing as we selected individuals among the sleep laboratory data and not in the general population. Therefore, they must have a sleep complaint and probably a sleep impairment to be included. Sleep disorder is one of the AD diagnosis criteria and is very frequent in depression of all types, but it is not mandatory. Thus, the sleep issue could be over-represented in our results (even so, we take it into account in the adjustment criteria). Another limitation of our polysomnography analysis is due to the subjects treated with benzodiazepine or antidepressant. Both could interfere with the sleep architecture. Despite adjustments performed, this parameter may influence our results. The third limit is related to a transversal study while we are investigating a chronic inflammation state. Nevertheless, CRP is presumed to be a reliable marker of chronicity in the case of LGI. The last limitation is the retrospective aspect of the data; therefore, these results need to be confirmed with a prospective study.

The strength of this study is due to the large sample of individuals selected and the analysis of both inflammatory state and sleep parameters in the context of MDD subtypes. This study could contribute to improving the knowledge of the inflammatory risks of MDD. Moreover, the PCA analysis allows us to design a physiopathology environment that confirms the cardiometabolic feature of AD and shows us new perspectives. Indeed, the low-grade inflammation state associated with atypical depression regarding the diagnosis criterion of hyperphagia allows us to see the nutritional parameter as a risk factor and could suggest an interventional study.

## 5. Conclusions

Our results confirm the cardiometabolic feature of the LGI group and its high prevalence in AD. As depression is a multifactorial psychiatric illness, the origin of the associated inflammatory state may also be multifactorial. Thus, markers of elevated hypoxemia could contribute to LGI, as well as the nutritional issues associated with AD. AD tends to last longer and has a high risk of suicide and anxiety disorders [72]. Considering the inflammatory state, as well as sleep and dietary patterns, could perhaps contribute to a better prognosis and lowered comorbidities. These results highlight the importance of a multifactorial approach for the diagnosis and treatment of severe depression.

## Figures and Tables

**Table 1:** Polysomnographic data: LGI and no-LGI groups.

	Median (P25–P75) Whole Sample (n = 765)	Median (P25–P75) Subjects without LGI (n = 538)	Median (P25–P75) Subjects with LGI (n = 227)	Wilcoxon Test *p*-Value	b_a1_ (ES)	*p*-Value Adjusted
Sleep latency (min)	33.0 (18.0–63.0)	32.5 (18.0–65.5)	34.0 (19.0–58.5)	0.879	3 (3.2)	0.341
Sleep efficiency (%)	79.2 (70.5–85.9)	79.3 (70.5–86.3)	79.1 (70.6–85.1)	0.470	0.2 (1.1)	0.837
Sleep period time (min)	445.0 (409.7–482.5)	445.0 (407.0–485.0)	445.0 (416.5–480.5)	0.687	−3.5 (5.3)	0.512
Total sleep time (min)	392.7 (344.5–431.0)	394.5 (345.0–435.3)	386.5 (344.0–425.0)	0.355	−7.6 (5.9)	0.196
% Stage 1	7.0 (4.7–9.7)	7.1 (4.7–9.6)	6.8 (4.7–10.0)	0.783	−0.2 (0.3)	0.492
% Stage 2	54.1 (47.1–61.1)	54.4 (47.1–61.1)	53.9 (46.5–61.1)	0.926	−1.1 (1.0)	0.295
% Stage 3	6.7 (1.0–14.0)	6.5 (1.1–13.6)	7.8 (0.8–15.4)	0.652	0.4 (1.0)	0.714
% REM	16.7 (12.3–20.9)	17.1 (12.8–21.4)	15.5 (10.9–19.7)	0.001	−1.6 (0.6)	0.006
REM latency (min)	88.5 (63.0–150.5)	83.0 (61.3–136.0)	105.0 (72.5–183.0)	<0.001	12.0 (5.3)	0.024
% wake after sleep onset	10.4 (5.8–17.2)	9.9 (5.8–16.8)	11.3 (5.7–18.1)	0.113	1.6 (0.8)	0.055
Number of awakenings	27 (19–39)	27 (19–39)	27 (19–41)	0.276	1.0 (1.3)	0.448
Micro-arousal index	9 (6–14)	9 (6–13)	9 (6–16)	0.159	1.0 (0.6)	0.105
Apnoea–hypopnoea index	3 (1–8)	3 (1–7)	3 (1–11)	0.013	0.2 (0.4)	0.568
Oxygen desaturation index	1 (0–4)	1 (0–4)	2 (0–6)	0.001	1 (0.3)	<0.001
Total time under 90% of SaO_2_ (min)	0.3 (0.0–11.0)	0.0 (0.0–5.7)	1.5 (0.0–21.0)	<0.001	1.5 (0.5)	0.001
PLMS index	1 (0–7)	2 (0–8)	1 (0–7)	0.019	−0.7 (0.4)	0.113

LGI = low-grade inflammation, REM = rapid eye movement sleep, SaO_2_ = oxygen saturation, PLMS = periodic limb movements during sleep. b_a1_ (ES): quantile regression coefficient adjusted (standard error). These coefficients are the difference of median adjusted for antidepressant therapy and benzodiazepine receptor agonists between “Subjects without LGI” and “Subjects with LGI”.

**Table 2:** Univariate analyses (n = 765).

Variables	Categories	%	Subjects without LGI	Subjects with LGI	*p*-Value Chi^2^	OR (CI 95%)	*p*-Value
Gender	Female (n = 416) male (n = 349)	54.4% 45.6%	49.8% 50.2%	65.2% 34.8%	<0.001	1 0.53 (0.38 to 0.73)	<0.001
Age (years)	<40 (n = 299) ≥40 (n = 466)	39.1% 60.9%	41.6% 58.4%	33.0% 67.0%	0.026	1 1.45 (1.04 to 2.00)	0.026
BMI (kg/m^2^)	<25 (n = 302) ≥25 (n = 463)	39.5% 60.5%	46.8% 53.2%	22.0% 78.0%	<0.001	1 3.12 (2.18 to 4.46)	<0.001
Antidepressant therapy	No (n = 455) Yes (n = 310)	59.5% 40.5%	61.5% 38.5%	54.6% 45.4%	0.076	1 1.33 (0.97 to 1.82)	0.076
Benzodiazepine receptor agonists	No (n = 599) Yes (n = 166)	78.3% 21.7%	79.0% 21.0%	76.7% 23.3%	0.472	1 1.15 (0.79 to 1.66)	0.473
Smoking	No (n = 576) Yes (n = 189)	75.3% 24.7%	77.3% 22.7%	70.5% 29.5%	0.045	1 1.43 (1.01 to 2.02)	0.046
Alcohol	No (n = 526) Yes (n = 239)	68.8% 31.2%	68.2% 31.8%	70.0% 30.0%	0.618	1 0.92 (0.66 to 1.29)	0.618
Caffeine	No (n = 181) Yes (n = 584)	23.7% 76.3%	23.1% 76.9%	25.1% 74.9%	0.540	1 0.89 (0.62 to 1.28)	0.540
Type 2 diabetes	No (n = 681) Yes (n = 84)	89.0% 11.0%	91.8% 8.2%	82.4% 17.6%	<0.001	1 2.40 (1.52 to 3.80)	<0.001
Dyslipidemia	No (n = 434) Yes (n = 331)	56.7% 43.3%	60.0% 40.0%	48.9% 51.1%	0.005	1 1.57 (1.15 to 2.15)	0.005
Hypertension	No (n = 488) Yes (n = 277)	63.8% 36.2%	68.0% 32.0%	53.7% 46.3%	<0.001	1 1.83 (1.33 to 2.52)	<0.001
Cardiovascular comorbidities	No (n = 697) Yes (n = 68)	91.1% 8.9%	92.6% 7.4%	87.7% 12.3%	0.030	1 1.75 (1.05 to 2.92)	0.031
Aspirin therapy	No (n = 710) Yes (n = 55)	92.8% 7.2%	93.1% 6.9%	92.1% 7.9%	0.607	1 1.17 (0.65 to 2.10)	0.607
OSAS	No (n = 487) With TO_2_ 90% < 10 min (n = 149) With TO_2_ ≥ 10 min (n = 129)	63.7% 19.5% 16.8%	65.4% 19.9% 14.7%	59.5% 18.5% 22.0%	0.046	1 1.02 (0.68 to 1.54) 1.65 (1.10 to 2.48)	0.048
Insomnia disorder	No (n = 186) Without short sleep duration (n = 397) With short sleep duration (n = 182)	24.3% 51.9% 23.8%	25.3% 51.1% 23.6%	22.0% 53.7% 24.3%	0.627	1 1.21 (0.82 to 1.78) 1.18 (0.75 to 1.85)	0.628
Sleep movement disorders	No (n = 631) Moderate to severe PLMs alone (n = 46) RLS alone or combined with PLMs (n = 88)	82.5% 6.0% 11.5%	82.2% 6.5% 11.3%	83.3% 4.8% 11.9%	0.671	1 0.73 (0.37 to 1.48) 1.04 (0.64 to 1.68)	0.673
EDS	No (n = 366) Yes (n = 399)	47.8% 52.2%	49.6% 50.4%	43.6% 56.4%	0.128	1 1.27 (0.93 to 1.74)	0.128
Depression severity	Mild to moderate (n = 549) severe (n = 216)	71.8% 28.2%	72.7% 27.3%	69.6% 30.4%	0.388	1 1.16 (0.83 to 1.63)	0.389
Depression subtype	OD (n = 596) AD (n = 169)	77.9% 22.1%	80.3% 19.7%	72.3% 27.7%	0.014	1 1.57 (1.09 to 2.24)	0.015
LGI	No (n = 538) Yes (n = 227)	70.3% 29.7%					
	Median (P25–P75)				Wilcoxon test		
Age (years)	43 (33–52)		42 (33–51)	45 (36–53)	0.071		
BMI (kg/m^2^)	26.6 (22.9–31.1)		25.5 (22.2–29.1)	30.5 (25.6–36.2)	<0.001		
CRP (mg/L)	1.6 (0.8–3.5)		1.1 (0.7–1.8)	5.2 (3.8–7.2)	<0.001		
ESS	11 (7–14)		11 (7–14)	12 (7–15)	0.431		
ISI	18 (15–21)		18 (14–21)	18 (15–21)	0.430		
BDI	12 (9–16)		12 (10–16)	13 (9–17)	0.445		

LGI = low-grade inflammation, BMI = body mass index, OSAS = obstructive sleep apnea syndrome, TO_2_ 90% = total time under 90% of SaO_2_, PLMs = periodic limb movements during sleep, RLS = restless legs syndrome, EDS = excessive daytime sleepiness, CRP = C-reactive protein, ESS = Epworth sleepiness scale, ISI = insomnia severity index, BDI = Beck depression inventory.

**Table 3:** AD_OD groups multivariate analyses.

Variables	Model 1 OR Adjusted (CI 95%)	*p*-Value	Model 2 OR Adjusted (CI 95%)	*p*-Value	Model 3 OR Adjusted (CI 95%)	*p*-Value	Model 4 OR Adjusted (CI 95%)	*p*-Value
MDD		0.007		0.007		0.045		0.047
OD	1		1		1		1	
AD	1.67 (1.15 to 2.41)		1.66 (1.15 to 2.41)		1.48 (1.01 to 2 2.18)		1.48 (1.01 to 2.18)	

Model 1 = Model adjusted for type 2 diabetes, dyslipidemia, hypertension, and cardiovascular comorbidities. Model 2 = model adjusted for type 2 diabetes, dyslipidemia, hypertension, cardiovascular comorbidities, and smoking. Model 3 = model adjusted for type 2 diabetes, dyslipidemia, hypertension, cardiovascular comorbidities, smoking, gender, age, and BMI. Model 4 = model adjusted for type 2 diabetes, dyslipidemia, hypertension, cardiovascular comorbidities, smoking, gender, age, BMI, and OSAS. BMI = body mass index, OSAS = obstructive sleep apnea syndrome. AD = MDD with atypical features. OD = MDD without atypical features.

**Table 4:** Variance explained by the principal components (PC1, 2, 3). The cumulative result (PC1, 2, 3) explain 0.439 of the model variances.

Component Characteristics			
	Eigenvalue	Proportion Var.	Cumulative
PC1	2.422	0.220	0.220
PC2	1.304	0.119	0.339
PC3	1.098	0.100	0.439

**Table 5:** Component loadings: each axis shows which variables are the most correlated. The sign (+/−) indicates if the correlation is positive or negative.

Component Loadings				
	PC1	PC2	PC3	Uniqueness
LGI	0.235	0.715	0.236	0.379
AD		0.584		0.624
CVD	0.326		0.686	0.403
Dyslipidemia	0.586			0.648
Diabetes	0.551			0.676
Smoking status			0.386	0.848
Sex			−0.587	0.420
BMI	0.554	0.569		0.332
Age	0.585			0.629
OSAS	0.518			0.698
Hypertension	0.691			0.519

Note. Applied rotation method is varimax.

**Table 6:** Estimated prevalence calculation of LGI in AD and OD. n(AD) = 164, n(OD) = 596, total MDD individuals n = 760.The χ^2^ test = 0.047. The effectiveness in each group is different from previous calculation due to the LGI range value adjustment.

		Non-LGI (0) LGI(1)		
AD = 1, OD = 0		0	1	Total
0	Observed	436	160	596
	%per line	73.2%	26.8%	100.0%
1	Observed	107	57	164
	%per line	65.2%	34.8%	100.0%
Total	Observed	543	217	
	%per line	71.4%	28.6%	100.0%

**Table 7:** Hypertension AD vs. OD, *p* = 0.065.

Tests χ^2^		
	Valeur	ddl
χ^2^	3.42	1
N	765	

## Data Availability

The data presented in this study are available on request from the corresponding author (the data are not publicly available due to privacy restrictions).

## Supplementary Materials

The following supporting information can be downloaded at: https://www.mdpi.com/article/10.3390/brainsci14090850/s1, File S1: Details of psychiatric and sleep assessment and additional calculation from statistical analysis (PCA and univariate analyses). References [73,74,75,76,77,78,79,80,81,82,83] are cited in the supplementary materials.

## Abbreviations

AD = MDD with atypical features, OD = MDD without atypical features, AHI = Apnea–Hypopnea Index, BDI = Beck Depression Inventory, BMI = body mass index, CVD = cardiovascular disease, CRP = C-reactive protein, DSM = Diagnostic and Statistical Manual of Mental Disorders, EDS = excessive daytime sleepiness, ESS = Epworth Sleepiness Scale, LGI = low-grade inflammation, ISI = Insomnia Severity Index, MDD = Major Depressive Disorder, MDI = Major Depressive Individual, ODI = Oxygen Desaturation Index, OSAS = obstructive sleep apnea syndrome, PCA = principal components analysis, PLMs = periodic limb movements during sleep, TO_2_ = total time under 90% oxygen saturation, REM = rapid eye movement sleep, RLS = restless legs syndromes, SaO_2_ = oxygen saturation, WD = Western diet.
