# Supplementary material for: Low-Grade Inflammation Associated with Major Depression Subtypes: A Cross-Sectional Study
Source: Brain Sci. 2024 Aug 23;14(9):850. doi: 10.3390/brainsci14090850 (PMC11430340; doi:10.3390/brainsci14090850)
Supplement: Supplementary file 1 [file brainsci-14-00850-s001.zip › brainsci-3145954-supplementary.pdf]

brain sciences

MDPI

Supplementary data

Low-Grade Inflammation Associated with Major Depression Subtypes: A Cross-Sectional Study

S1. Medical and psychiatric assessment of participants

S1.1 Psychiatric self-questionnaire:

The Beck Depression Inventory (abbreviated 13-item BDI) is used to investigate the presence of depressive symptoms. The 13 items of this scale can be scored from 0 to 3, which means that the total score can vary from 0 to 39. Depressive symptoms are considered absent with a score of 0 to 4, mild with a score of 5 to 7, moderate with a score of 8 to 15, and severe with a score above 16 [74].

The Epworth Sleepiness Scale is used to investigate excessive daytime sleepiness. The 8 items of this scale assess daytime sleepiness in different daytime situations and can be scored from 0 to 3, so that the total score can vary from 0 to 24. A score above 10 indicates excessive daytime sleepiness [75].

The Insomnia Severity Index is used to investigate the presence of insomnia symptoms. The 7 items of this index can be scored from 0 to 4, so that the total score can vary from 0 to 28. Insomnia symptoms are considered absent if the score is 0 to 7, subclinical if the score is 8 to 14, moderate if the score is 15 to 21, and severe if the score is over 22 [76].

S1.2 Sleep assessment and examination.

Sleep history : The sleep laboratory psychiatrist conduct an interview for all individuals admitted to perform a complete inventory of their sleep-related complaints, including sleep habits, severity of self-reported insomnia complaints (difficulty falling asleep, repeated nocturnal awakenings, early morning awakening and unrefreshing sleep), symptoms related to sleep apneas (snoring and self-reported apneas), symptoms related to restless legs syndrome (impatience of the legs with or without abnormal sensations: aggravated by rest, partially or temporarily relieved by movement and increased during the evening or night) and abnormal nocturnal movements (periodic limb movements).

S1.3. Polysomnography.

Participants had a polysomnographic recording from which data are collected for analysis. The data are Sleep latency, efficiency and period time, total sleep time (stage 1, 2,3), % of rapid eyes movements (REM) and REM latency, % of awake after sleep onset, number of awakenings, microarousal index , Apnoea-hypopnoea index, oxygen desaturation index , total time under 90% of oxygen saturation (min), periodic limb movement during sleep (PLMS).The polysomnographies performed are compliant with the recommendations of the American Academy of Sleep Medicine [38].

Patients go to bed between 22:00 - 24:00 and get up between 06:00 - 8:00, following their usual schedule. During bedtime, patients lie in bed with the lights off. Daytime naps are not permitted. The polysomnographic set-up applied is as follows: 2 electro-oculogram channels, 3 electroencephalogram channels, 1 submental electromyogram channel, 1 electrocardiogram, thermistors to detect oronasal airflow, 1 microphone to record respiratory sounds and snoring, piezoelectric sensors as well as strain gauges to measure thoracic and abdominal respiration and electrodes measuring leg movements. Polysomnographic recordings are visually scored by specialized technicians according to the criteria of the American Academy of Sleep Medicine [77]. Apneas are scored

if the decrease in airflow is ≥ 90% for at least 10 seconds while hypopneas are scored if the decrease in airflow is ≥30% for at least 10 seconds with a decrease in oxygen saturation of 3% or followed by microarousal [78]. The apnea-hypopnea index corresponds to the total number of apneas and hypopneas divided by the sleep period in hours. Obstructive Sleep Apnea (OSA) is considered absent when the apnea-hypopnea index is <5/hour, mild when the apnea-hypopnea index is ≥5/hour and <15/hour and moderate to severe when the apnea-hypopnea index is ≥15/hour [79]. Periodic limb movements are scored according to the following criteria: 1) a duration between 0.5 and 10 seconds, 2) an interval between 5 and 90 seconds from the onset of limb movement, and 3) the movements are part of a sequence of at least 4 consecutive movements meeting the preceding criteria [80]. The index of periodic limb movements corresponds to the total number of periodic limb movements divided by the sleep period in hours. Periodic limb movement syndrome is considered present when the periodic limb movement index is ≥15/hour [81]. In addition, diagnoses of restless legs syndrome are made according to the diagnostic criteria of the International Restless Legs Syndrome Study Group [82]. Finally, diagnoses of insomnia disorders are made according to the diagnostic criteria of the American Academy of Sleep Medicine Work [83] while sleep deprivation is defined as sleep duration <6 hours [84].
